# ProAKAP4 concentration in fresh canine semen and its correlation with motility parameters

**DOI:** 10.1016/j.vas.2025.100455

**Published:** 2025-04-17

**Authors:** Djemil Bencharif, Rédha Belala, Nora Mimoune, Dolorès Le Couazer, Hamed Farnia

**Affiliations:** a Department of Biotechnology and Pathology of Reproduction, ONIRIS, Nantes, France; b INSERM, Nantes Université, CHU Nantes, Center for Research in Transplantation and Translational Immunology, UMR 1064, F-44000 Nantes, France; c Biotechnologies Laboratory Related to Animal Reproduction (LBRA), Institute of Veterinary Medicine, Saad Dahleb Blida University 1, Blida, Algeria; d Biotechnologies Platform for Animal Medicine and Reproduction (BIOMERA), Saad Dahleb Blida University 1, Algeria; e Higher National Veterinary School, Algiers, Algeria

**Keywords:** AKAP4 precursor, Dog, Sperm, CASA motility, Correlation

## Abstract

This study aimed to evaluate proAKAP4concentrations in canine fresh semen and to determine their correlation with sperm motility and fertility parameters. For this purpose, 26 ejaculates from 18 dogs were analyzed. Motility and kinetic parameters of the sperm-rich fractions were assessed by a computer assisted semen analyzer (Hamilton Thorne HT-IVOS II, USA). ProAKAP4 concentrations were measured (containing either the spermatic, urethral, or prostatic fraction) using the Dog 4MID® kit (4BioDx, France). In the spermatic fractions, on average, the proAKAP4 concentration was 17.08 ng/10^6^spermatozoid (spz) with a total motility of 75.92 % and progressive motility of 58.08 %. Spearman test showed a positive correlation between proAKAP4 concentrations and the total motility (r = 0.584; p = 0.08), progressive motility (r = 0.6336; p = 0.0005), and the velocity parameters: VAP (r = 0.4178; p = 0.0337), VSL (r = 0.3919; p = 0.0477) and mainly VCL (r = 0.5266; p = 0.0057). For fertility monitoring, 5 ejaculates were used to perform artificial insemination (AI). The mean proAKAP4 concentration was 25.91 ng/10^6^spz with an average total motility of 74.4 % and progressive motility of 53 %. Four female dogs were pregnant with litter sizes ranging from 4 to 11 puppies. To conclude, this is the first study presenting the significant correlation between proAKAP4 concentration and sperm motility parameters in dogs. Further investigations with a large number of animals are recommended to test the correlation between proAKAP4 and fertility parameters.

## Highlights

• First study linking proAKAP4 concentration with sperm motility in dogs.
• Positive correlation between proAKAP4 and total/progressive sperm motility.
• Significant association between proAKAP4 and sperm velocity parameters (VAP, VSL, VCL).
• Successful artificial insemination with proAKAP4 concentration linked to fertility.

## 1 Introduction

Functional sperm quality markers to predict male fertility have been actively investigated. Among them, proAKAP4 (the precursor of AKAP4: A-Kinase Anchoring Protein 4), is a 97 kDa protein, present at the round spermatid stage (Dordas-Perpinyà et al., 2022a). This protein is transported in the fibrous sheath of the flagellum during its development. At the condensed nucleus spermatid stage, proAKAP4 is cleaved and gives the mature AKAP4 protein (Brown et al. 2003); (Le Couazer and Bencharif, 2021). ProAKAP4 and its active form, AKAP4, are expressed in a variety of mammalian species, and they are highly conserved (∼70 %) across animal kingdom (Dordas-Perpinyà et al., 2022b). They are found along the entire length of the principal piece of the flagellum in rat (Carrera et al., 1994), bull (Bastan & Akcay, 2021), equine (Blommaert et al., 2019), boar (Sergeant et al., 2019), and camel (Malo et al., 2021) spermatozoa.

AKAP4 is implicated in sperm motility regulation. It is phosphorylated by tyrosine kinases, initiating a cascade of protein phosphorylation associated with motility (Carrera et al., 1996; Pereira et al., 2017). Specifically, AKAP4 exerts an impact on the specificity of the transduction signal and metabolic processes that support sperm motility, hypermobility, and capacitation (Dordas-Perpinyà et al., 2022b). In AKAP4-knockout mice, spermatozoa exhibit flagellar disorganization, which renders them immotile and consequently infertile (Malo et al., 2021). In humans, absent or low expression levels of AKAP4 and proAKAP4 are associated with decreased motility and fibrous sheath dysplasia syndrome (Moretti et al., 2007; Delehedde et al., 2019b). AKAP4 is clearly down regulated in infertile patients with asthenozoospermia (Jumeau et al., 2018a; 2018b). In women, previous data reported that proAKAP4 amounts are negatively correlated with the abortion rate in intrauterine inseminated patients. Similarly, AKAP4 levels are positively correlated with embryo quality, improved embryo freezing outcomes, implantation rates, and pregnancy rates in humans (Jumeau et al., 2018a).

In dogs, previous research identified the presence of ProAKAP4 and AKAP4 in the fibrous sheath of the spermatozoa extracted from the testicles and in the spermatic fraction of the ejaculate (not in urethral nor in prostatic fractions) (Le Couazer & Bencharif, 2021). In this study, the objective was to determine the proAKAP4 concentration in fresh dog sperm and to evaluate its correlation with sperm motility and fertility parameters.

## 2 Material and methods

### 2.1 Animals

In this study, 26 ejaculates (urethral, spermatic and prostatic fractions) from 18 healthy dogs of different breeds (Smooth-faced Pyrenean Shepherd, Dutch Shepherd, Labrador Retriever, Continental Bulldog, Belgian Tervuren, Newfoundland, Belgian Malinois, Leonberger, Australian Shepherd, Hovawart, Bobtail), with an age ranging from 2 years and 8 months to 11 years were collected at the Artificial Insemination Center (CIAC) of the National Veterinary School of Nantes (ONIRIS), to perform either a spermogram or artificial insemination (AI) of the females. These dogs were belonging to some private owners who accepted to support our experimental design and to follow the full process of the study.

Among the 18 males, 13 were sampled once, 2 were sampled twice, and 3 were sampled three times.

All dogs were manually sampled by the same operator (in order to standardize handling and to minimize bias), using an artificial vagina heated to 37 °C and lubricated with a non-spermicidal solution. The samples were taken in the presence of a female in heat. An assistant was present to collect the 3 fractions of the ejaculate in different tubes which were placed in a water bath at 37 °C.

All animal handling process met the requirements of the Oniris Ethics Committee.

### 2.2 Motility assessment

Immediately after collection, the semen quality was analyzed. Mass motility, scored on a scale of 0 to 5, was assessed under a hot-stage optical microscope. Semen concentration was measured using a spectrophotometer previously calibrated for canine semen (SpermaCue®, Minitube®, Germany) with a slide standardized to 533 ± 5 × 10^6^spz/mL.

Automated sperm motility analysis was performed using the Hamilton-Thorne IVOS II image analyzer. The IMSI Strict™ software allowed the acquisition of the following parameters: Total motility (%), progressive motility ( %), Curvilinear Velocity (VCL, µm/s), Amplitude of Lateral Head Displacement (ALH, µm), Straight Line Velocity (VSL, µm/s), and Average Path Velocity (VAP, µm/s).

### 2.3 ProAKAP4 analysis

In the experiment, 58 samples were stored in a cryotube and placed at −20 °C in a domestic freezer for 6 months. This corresponded to 100 μL of 26 spermatic fractions, 100 μL of 16 urethral fractions and 100 μL of 16 prostatic fractions from the same dogs.

In the 58 samples, the ProAKAP4 assay was performed using the ELISA kit developed by the 4 BiodXLaboratory: Dog 4MID Kit (4VDX-18K5, France). This is a complete ELISA kit including a pre-coated microplate, the reagents and buffers necessary for the detection and quantification of proAKAP4 in dog semen. The calibration range was first carried out in order to obtain a standard curve. The spermatozoa were first denatured using a lysis buffer specific to canine species, which allowed the extraction of proteins from this cell (spermatozoa).

ELISA protocol was then carried out: the prepared sperm samples were placed in the wells containing the anti-proAKAP4 antibody. Then, the use of the detection antibody allowed the revelation of the immune complexes. The optical density of each well was determined using a spectrophotometer set at 450 nm. The standard curve allowed the determination of proAKAP4 concentration in ng/mL in the samples (expressed in ng/10^6^ of spz by dividing the proAKAP4 concentration obtained (in ng/mL) by the sperm concentration of the sample so as not to have any bias from the concentration of the sperm used).

### 2.4 Fertility monitoring

To follow-up fertility, 5 ejaculates (from 5 dogs) among the 26 initial ejaculates were used for AI.

Heat monitoring was performed on all bitches before AI. Vaginal smears were carried out using Harris Schorr staining (Diagnoestrus®, RAL reagents). Once the vaginal smear showed an image of estrus (clean smear background, high cell density, predominance of keratinized cells), blood progesterone (P4) levels were assessed every 48 h. Blood samples were taken on heparinized tubes, and then centrifuged to recover the plasma. P4 rates were measured with the Mini VIDAS® (Biomerieux, France). As soon as the level reached or exceeded 10 ng/mL, AI was performed and repeated 48 h later (in 3 females, intrauterine; in 1, intravaginal; in 1, intrauterine using laparotomy).

Intrauterine inseminations were performed under vaginoscopy, after visualization of the cervix, the latter was catheterized by means of a suitable sterile and single-use urinary catheter, into the uterus where only the sperm fraction was injected. On the other hand, in the case of artificial insemination at the vaginal level, the Osiris type probe was introduced up to the bottom of the vagina and the 3 fractions of the semen, namely urethral, ​​spermatic and prostatic, were injected into the vagina in the order of their collection. The probe was then washed and sterilized in the autoclave to be reused again. Finally, for intrauterine insemination by laparotomy, the female dog was under general anesthesia, and inseminated 48 h after the progesterone level reached or exceeded 10 ng/mL. The uterine horns were punctured and catheterized with a single-use venous catheter.

Pregnancy diagnosis was performed 15 to 30 days after AI by an ultrasound scanner equipped with a 12.5 MHz probe. The diagnosis was negative if no fetus was visualized 30 days post AI.

### 2.5 Statistical analysis

Statistical study was performed using GraphPad Prism 8.1.2 software (San Diego, CA, USA). The Spearman Rank Correlation test was used to evaluate the correlation between proAKAP4 concentrations and motility parameters. Correlations were considered as significant when *P* < 0.05 (*), *P* < 0.01 (**) and *P* < 0.001 (***). Data were expressed as mean Standard deviation (SD).

**Fig. 1:**
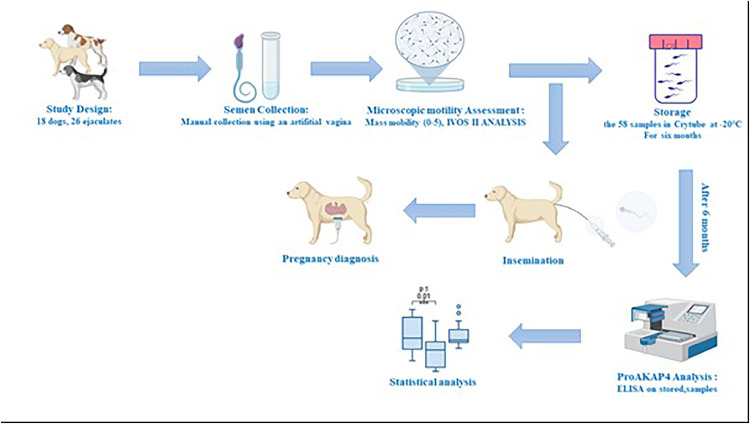
Experimental design summary.

Fig. 1. Summarize the full experimental design.

## 3 Results

### 3.1 ProAKAP4 concentrations in the 3 ejaculate fractions

**Fig. 2:**
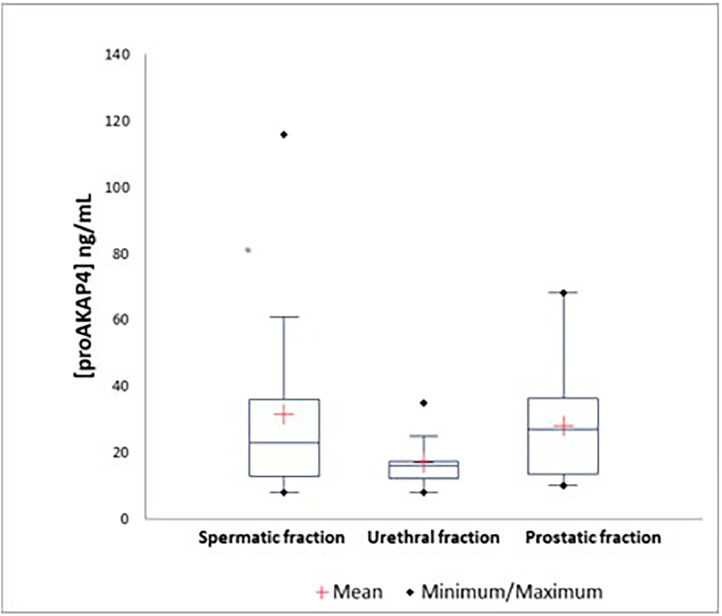
Box plot of proAKAP4 concentration in the 3 ejaculate fractions of 58 samples.

The summary of descriptive statistics concerning the concentration of proAKAP4 in ng/mL in the 3 fractions of 26 ejaculates are presented in Table 1, Fig. 2.

**Table 1:** ProAKAP4 concentration in the 3 fractions of 26 ejaculates.

Fraction	[ProAKAP4] ng/mL	Maximum	Minimum
**Urethral fraction**	19.40±8.16	51.20	8.48
**Spermatic fraction**	31.38±26.83	116.20	8.53
**Prostatic fraction**	28.19±13.44	68.48	10.70

Fig. 2 showed the proAKAP4 concentration in ng/mL of each ejaculate fraction (n = 26).

Error bars overlap. Thus, it seemed that there is no significant difference between the mean ProAKAP4 concentrations in the three fractions of dog ejaculate.

**ProAKAP4 concentration in spermatic fraction and the correlation with motility parameters**

Descriptive statistics regarding proAKAP4 concentration in ng/10^6^spz and motility parameters of spermatic fraction samples (n = 26) are presented in Table 2. On average, proAKAP4 concentration was 17.08 ng/10^6^ spermatozoa, total motility was 75.92 %, and progressive motility was 58.08 %.

**Table 2:** ProAKAP4 concentrations and motility parameters of spermatic fraction (n = 26).

	[ProAKAP4] (ng/10^6^ spz)	Total motility (%)	Progressive motility (%)
**Mean±SD**	17.08±13.03	752±21.05	58.08±23.91
**Maximum**	47.36	96	89
**Minimum**	2.37	28	15

**Fig. 3:**
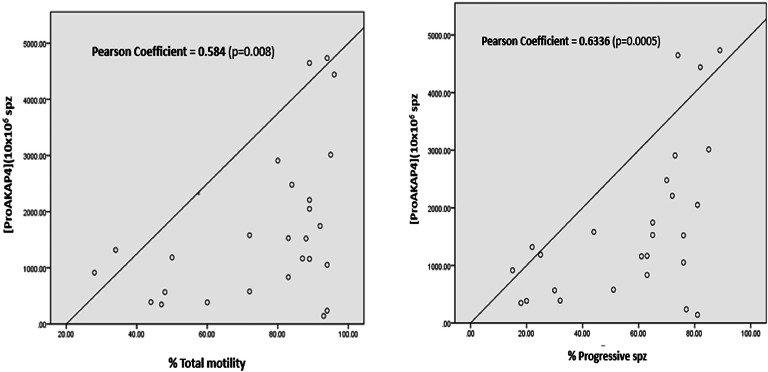
ProAKAP4 concentration and motility parameters correlation in spermatic fractions (n = 26).

According to the results shown in Table 4 and Fig. 3, proAKAP4 concentrations are positively correlated with total motility (r = 0.584 and p-value=0.0080(**)), and with progressive motility (r = 0.6336 and p-value=0.0005 (***)).

**Table 3:** Fertility data.

Dog	[ProAKAP4] (ng/10 × 10^6^ spz)	Total motility (%)	Progressive motility (%)	Pregnancy Diagnostic	Number of puppies
1	3.85	60	20	Not inseminated	0
2	24.80	47	28	+	11
3	15.28	83	65	+	8
4	10.52	94	76	+	9
5	15.22	88	76	+	4

**Table 4:** Results of sperm motility parameters, proAKAP4 concentration and sperm concentration on the sperm fractions tested.

Individual	ProAKAP4 concentration (ng/10.10^6 sperm)	VAP (μm/s)	VSL (μm/s)	VCL (μm/s)	ALH (μm)
Dog 1	3,85	63,6	53,9	108,7	6
Dog 2	5,79	95,4	82,4	137,8	6
Dog 2	8,34	106,9	96,2	146,6	5,8
Dog 2	15,81	83,8	73,7	131,6	6,3
Dog 3	24,80	123,3	112,9	160,3	5,6
Dog 4	30,13	152	141,4	180,6	5,3
Dog 4	3,90	77	98,4	102,6	5,7
Dog 4	20,50	136,8	130,7	159,4	4,6
Dog 5	15,28	134,4	118	175,8	6,7
Dog 6	10,52	143	129,2	173	5,3
Dog 7	2,37	115	101,5	166,9	6,6
Dog 8	9,15	74,9	66,1	114,2	5,7
Dog 8	11,86	82,8	67,8	130,2	6,4
Dog 9	3,48	56,7	46,1	91,2	5,4
Dog 9	17,45	112,8	93,3	154,7	6,2
Dog 9	13,18	81,2	75,2	111,8	4,7
Dog 10	44,42	130,3	117,8	161,8	5,5
Dog 11	11,66	116,2	104,3	148,9	5,1
Dog 12	22,09	116,1	103,5	157,8	5,7
Dog 13	11,59	99,8	83,9	158,3	6,7
Dog 14	47,36	95	75,1	191,7	7,8
Dog 14	46,48	149	136,1	184,6	5,5
Dog 15	14,2	152,2	139,3	191,9	6,1
Dog 16	15,22	151,4	139,4	182,7	6,3
Dog 17	5,68	90,4	78,9	139,1	6,7
Dog 18	29,09	143,3	132,4	181,5	6,3

ProAKAP4 concentration (ng/10^6^spz) is weakly correlated with VAP (r = 0.4178 and p-value=0.0337 (*)), and with VSL (r = 0.3919 and p-value=0.0477 (*)), correlated with VCL (r = 0.5266 and p-value=0.0057 (**)) and is not correlated with ALH (r=−0.03188 and p-value=0.8771 (NS)) (Table 4 andFig. 4).

**Fig. 4:**
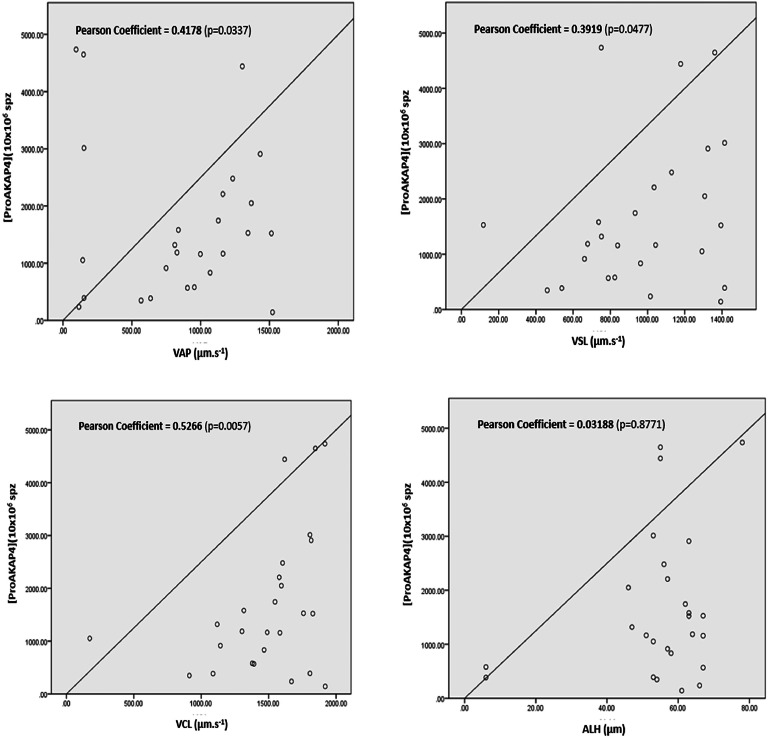
ProAKAP4 concentration and kinetic parameters correlation in spermatic fractions (n = 26).

### 3.2 Fertility monitoring results

In the study of fertility, no statistical test could be performed due to the small number of inseminated females and the use of completely different insemination techniques (in this case 3). We just aimed to determine the level of ProAKAP 4 in the sperm used for inseminations.

Among the 5 ejaculates used to perform AI, the mean concentration of proAKAP4 was 25.91 ng/10^6^ spermatozoa, the mean total motility was 74.4 % and progressive motility was 53 %. On the other hand, 1 ejaculate was not used for artificial insemination the next day, since the motility of the semen was considered insufficient less than 30 % motile sperm (Table 3).

Among the 4 AI performed on site with fresh semen, all inseminated bitches were found to be pregnant. Regarding the sending of refrigerated semen, the female dog was ultimately not inseminated due to poor sperm quality upon receipt. The litter size of the inseminated females ranged from 4 to 11 puppies, in accordance with the breed of animals. Due to the low number of inseminations, there is no clear correlation between ProAKAP4 levels and the number of puppies born per female dog.

## 4 Discussion

One of the primary limitations of the proAKAP4 analysis is its duration, which takes approximately eight hours. This timeframe may hinder its use in real-time semen evaluation before artificial insemination. To enhance its practical applicability, future research should focus on optimizing the testing protocol, potentially by developing rapid detection assays or integrating high-throughput analytical techniques. Additionally, exploring point-of-care diagnostic tools or predictive modeling based on partial data could serve as viable alternatives to provide preliminary insights while awaiting full results.

In our study, ProAKAP4 concentrations were only expressed in ng/10 × 10^6^spz so as not to have any bias from the sperm concentration of the ejaculate. This allowed for an objective comparison of proAKAP4 levels since proAKAP4 is contained in spermatozoa.

Because we had to have, 78 samples (divided into 26 urethral fractions, 26 sperm-rich fractions and 26 prostatic fractions), 20 samples were not taken into consideration, and since sometimes, the male was stressed and did not give a prostatic fraction. In some cases, we had dogs that gave only the spermatic fraction. Therefore, a total of 58 samples were included in this experiment.

Data showed that there is no significant difference between the mean proAKAP4 concentrations in the 3 fractions of the dog ejaculates. However, the results of a previous experiment (Le Couazer & Bencharif, 2021) indicated that proAKAP4 was not present in the urethral and prostatic fractions of the ejaculate. It can be due to the sampling technique because if sperm contaminates the urethral or prostatic fractions, there may be ProAKAP4 in the samples. In addition, during sampling, we are not safe from this contamination since the tubes are changed when there is a modification in color for the passage from the urethral fraction to the spermatic one or from the spermatic fraction to the prostatic one. There may be a little sperm in the fractions. This could be solved by the centrifugation of both the urethral and prostatic fractions to remove the sperm that will remain in the pellet; therefore, the urethral or prostatic fluid will not contain sperm, and consequently there will be no ProAKAP4.

A highly significant correlation was found between proAKAP4 concentrations and the percentage of progressive spermatozoa, in agreement with previous reports in mammals (Dordas-Perpinyà et al., 2022a; Jumeau et al., 2018a). ProAKAP4 concentrations were also strongly correlated with total motility and VCL, weakly correlated with VAP and VSL, and not correlated with ALH. These results are of great importance because they affirm that proAKAP4 is a marker of sperm quality in dogs since it is correlated with the main parameters currently used to evaluate semen (Belala et al., 2024). These findings align with those detected previously in humans, bulls, and boars (Sergeant et al., 2016; Singh et al., 2019; Bastan & Akcay, 2021). In the same context, Dordas-Perpinyà et al. (2022b) indicated that proAKAP4 could be a good biomarker of semen quality in stallions due to its correlation with progressive motility, as well as its expression in more mature spermatozoa.

Finally, in this experiment, dog number 9 was sampled 3 times. The first two samples were taken >2 months apart (therefore a spermatogenesis cycle took place). The first sperm sample had a proAKAP4 concentration of 17.39 ng/10.10^6^ sermatozoa while the second had a rate of 34.91 ng/10.10^6^ spermatozoa. Thus, even if this only concerned one animal, it can be estimated that the proAKAP4 concentrations can vary according to the different spermatogenesis cycles in the same dog.

Therefore, it would be interesting to use proAKAP4 as a mean for monitoring animal reproductive career. Furthermore, proAKAP4 rates in mice sperm were affected by factors such as exposure to tobacco or diet (Delehedde et al., 2019a). It reflects the environmental factors to which the animal is subjected. Thus, we could use proAKAP4 concentration as a marker to know when an individual is ready to be put back into reproduction after exposure to a treatment or a disease.

The semen of dog 1, which was not inseminated due to poor sperm quality upon receipt, showed low proAKAP4 concentrations. We suggest that proAKAP4 concentration in the spermatic fraction is indeed indicative of the sperm viability. However, it is also possible that the conditions of semen transport were not optimal for its preservation since the shipment was carried out under cold conditions at +4 °C. Particularly in this case, 2 facts should highlighted: The first is that we can predict the sperm quality if we want to refrigerate the semen at +4 °C but the dosage of the ProAKAP4 is not fast, it takes at least 8 h to get the results which is quite long. We can possibly wait for the data by refrigerating the sperm. If the results of ProAKAP4 are high, we can ship the semen with a 24-hour delay or perform artificial insemination on site. This is why it would be interesting to perform this analysis 24 h before sending the samples in relation to the progesterone levels. Secondly, with CASA, we cannot predict whether the sperm is able to withstand refrigeration unless the motility values are very good (motility > 80 % and progressive spermatozoa > 40 %). Thus, with a rate of progressive motility around 20 %, there is no certainty, even though we cannot conclude on a single sample.

Regarding the other inseminated female dogs, all were pregnant with variable concentrations of proAKAP4 depending on the sperm used. All the semen used showed proAKAP4 concentrations in the spermatic fraction greater than 10 ng/10 × 10^6^ spermatozoa. In fact, it was revealed that the average concentration of this fraction was 17.08 ng/10 × 10^6^ spermatozoa. Therefore, it seemed that from the threshold of 10 ng/10 × 10^6^ spermatozoa, the chances of success in insemination are high. Nevertheless, it would be interesting to test this hypothesis on a large number of animals in order to verify whether the quantity of proAKAP4 is positively correlated with a better implantation rate in AI and a better pregnancy rate.

All the inseminated female dogs were pregnant, but as in any insemination, the female effect should not be neglected. Indeed, a negative pregnancy diagnosis does not necessarily mean that the sperm used is of poor quality: optimization of the choice of the moment of insemination and the intrinsic fertility of the female play a crucial role (Derdour et al., 2022).

Furthermore, the results obtained during artificial inseminations in this study are not significant, but on the other hand, they show a trend which would seem to evolve according to the level of ProAKAP4 measured in the sperm before the male is put into reproduction.

Regarding litter size, the female that gave birth to the largest number of puppies was inseminated with semen containing an average concentration of 24.80 ng/10 × 10^6^spz of proAKAP4 (the highest concentration in this experiment). Thus, it seems that the higher proAKAP4 concentration is associated with a total and progressive motility which are very good, and constitute one of the factors influencing fertility and in particular an increase in the number of puppies. This has already been demonstrated in boars (Sergeant et al., 2019). This correlation was not studied in our experiment. However, it would be interesting to test this hypothesis on a large number of samples in order to optimize the statistical tests.

## 5 Conclusions

Although proAKAP4 has been largely described in other mammals, this research study is the first to focus on the relationship between proAKAP4 concentration and the motility parameters in fresh canine semen. Data allowed highlighting significant correlations between the dosage of proAKAP4 in the sperm fraction of fresh semen and the motility parameters. ProAKAP4 concentration is indeed a biomarker of sperm quality because it is a reserve of functional motility: it is the precursor of AKAP4, itself an essential protein in the coordination of the sperm motility transduction signal. No clear correlation emerged concerning the proAKAP4 levels in the semen and the litter size of the inseminated females but it seemed that proAKAP4 concentration tended to be correlated with AI success rate. On the other hand, in terms of litter size, there was no correlation, even though the inseminated bitches gave birth to a number of puppies considered average for the breed. In view of the results obtained, the authors suggest that further investigations on a large number of identified dogs, a model for predicting semen fertility could be developed using a system of threshold concentrations of proAKAP4 for which the chances of success in AI and of obtaining a large litter size are optimal. The development of a technique that allows the dosage of ProAKAP4 quickly enough so that the seeds can be sent as quickly as possible after the samples are taken is highly recommended. While this study primarily serves as a baseline investigation into proAKAP4 levels, its findings may hold translational potential for veterinary practice. Further research should explore how these biomarkers can be integrated into routine diagnostic protocols, aiding in the assessment of reproductive health and fertility management in clinical settings. Additionally, longitudinal studies evaluating the correlation between proAKAP4 levels and reproductive outcomes could enhance its applicability in patient monitoring. If validated through larger-scale trials, this biomarker may serve as a valuable tool for veterinarians, offering a more precise and early assessment of reproductive function. Future work should also focus on refining reference ranges, developing standardized testing protocols, and assessing the feasibility of incorporating proAKAP4 analysis into routine veterinary diagnostics. Future research should focus on the development of rapid testing methods, such as point-of-care immunoassays or biosensors, that could significantly reduce turnaround time. Advances in microfluidic technologies or the integration of portable diagnostic tools could potentially enable real-time or near-real-time evaluation of proAKAP4 levels at the time of semen collection. Until such technologies are available, proAKAP4 may be best suited for retrospective analysis, semen-banking decisions, or for research and quality control purposes rather than same-day clinical use.

## Funding

This research did not receive any specific financial support from public, commercial, or not-for-profit sectors.

## CRediT authorship contribution statement

**Djemil Bencharif:** Writing – review & editing, Writing – original draft, Visualization, Validation, Supervision, Resources, Project administration, Methodology, Investigation, Funding acquisition, Formal analysis, Data curation, Conceptualization. **Rédha Belala:** Writing – review & editing, Writing – original draft, Validation, Resources, Methodology, Formal analysis, Data curation. **Nora Mimoune:** Writing – review & editing, Writing – original draft, Visualization, Validation, Project administration, Investigation, Formal analysis, Data curation. **Dolorès Le Couazer:** Writing – review & editing, Writing – original draft, Visualization, Validation, Software, Resources, Methodology, Investigation, Funding acquisition, Formal analysis, Data curation. **Hamed Farnia:** Writing – review & editing, Writing – original draft, Visualization, Supervision, Project administration, Funding acquisition, Formal analysis, Data curation, Conceptualization.

## Declaration of competing interest

The authors declare the following financial interests/personal relationships which may be considered as potential competing interests:

Bencharif Djemil reports was provided by Oniris Nantes - Chantrerie Campus. Bencharif Djemil reports a relationship with Oniris Nantes - Chantrerie Campus that includes: employment. No Anything If there are other authors, they declare that they have no known competing financial interests or personal relationships that could have appeared to influence the work reported in this paper.
